# A novel ferroptosis-related gene signature for prognostic prediction of patients with lung adenocarcinoma

**DOI:** 10.18632/aging.203140

**Published:** 2021-06-11

**Authors:** Jingjing Jin, Chuan Liu, Shanshan Yu, Lingyi Cai, Andriamifahimanjaka Sitrakiniaina, Ruihong Gu, Wenfeng Li, Fangfang Wu, Xiangyang Xue

**Affiliations:** 1 Department of Microbiology and Immunology, Institute of Molecular Virology and Immunology, School of Basic Medical Sciences, Wenzhou Medical University, Wenzhou, China; 2 China Medical University, Shenyang, China; 3 Department of Chemoradiation Oncology, The First Affiliated Hospital of Wenzhou Medical University, Wenzhou, Zhejiang, China; 4 Department of Emergency, The Second Affiliated Hospital and Yuying Children’s Hospital, Wenzhou Medical University, Wenzhou, China

**Keywords:** ferroptosis, FRGs, lung adenocarcinoma (LUAD), prognosis

## Abstract

Background: Lung adenocarcinoma (LUAD) is a heterogeneous disease characterized by high mortality and poor prognosis. Ferroptosis, a newly discovered iron-dependent type of cell death, has been found to play a crucial role in the development of cancers. However, little is known about the prognostic value of ferroptosis-related genes (FRGs) in LUAD.

Methods: In the present study, RNA-seq transcriptome data of LUAD patients were obtained from The Cancer Genome Atlas (TCGA) database. Cox regression analysis was used to construct a multigene signature. Kaplan–Meier survival and receiver operating characteristic (ROC) curves were utilized to assess the prognostic prediction efficiency of the constructed survival model. LUAD patients from the GSE30219 dataset were used for validation.

Results: We found 46 differentially expressed FRGs between LUAD and adjacent normal tissues. Via univariate and multivariate Cox regression analyses, 5 differentially expressed FRGs were identified as being highly correlated with LUAD. Patients were divided into low- and high-risk groups according to the risk score. We found that the overall survival (OS) of patients in the high-risk group was significantly worse than that of their low-risk counterparts. (*P* < 0.0001 in the TCGA dataset and *P* = 0.044 in the GSE30219 cohort). In addition, gene set variation analysis (GSVA) of the tumor microenvironment of the two groups may explain the different survival of LUAD patients.

Conclusions: Our study identified a novel FRG signature that could be used to evaluate and predict the prognosis of LUAD patients, which might provide a new therapeutic target for the treatment of LUAD patients.

## INTRODUCTION

Lung cancer is one of the leading causes of cancer-related death worldwide and is characterized by high mortality and poor prognosis [1, 2]. More than one-quarter (27%) of all cancer deaths were due to lung cancer in 2015 [3, 4]. Clinically, non-small cell lung cancer (NSCLC) accounts for most of the diagnosed cases of LUAD, and the common histologic type of NSCLC is responsible for ~40% of all lung cancer cases [5]. At present, the main clinical treatments for LUAD include surgical resection, chemotherapy, and radiation [4]. In recent years, although some progress has been made in terms of medical treatments, only 15% of LUAD patients achieve 5-year survival [6]. Numerous studies have demonstrated that LUAD is a highly heterogeneous disease with distinct genetic and transcriptomic characteristics among individual patients [3], and prognostic prediction of LUAD remains challenging. Therefore, it is urgent to identify novel prognostic gene signatures that can be used to make prognostic predictions and can serve as new therapeutic targets for the treatment of LUAD patients.

Emerging evidence has shown the crucial role of ferroptosis in the regulation of the growth and metastasis of cancers, which suggests its great potential for cancer therapy and prognosis prediction [7–9]. Ferroptosis, an iron-catalyzed type of regulated cell death, is characterized by the accumulation of excessive polyunsaturated fatty acid (PUFA) peroxidation products to a lethal level [10]. Distinct from traditional apoptosis, necrosis or autophagy, ferroptosis is a novel regulated cell death mechanism that serves as a nexus among metabolism, redox biology, and disease [8, 11]. Hua Yuan et al. [12] found that CDGSH iron sulfur domain 1(CISD1) in hepatocellular carcinoma cells can inhibit erastin-induced ferroptosis by protecting against iron-mediated mitochondrial lipid peroxidation. BoyiGan and colleagues demonstrated that overexpression of the deubiquitinase ovarian tumor (OTU) family deubiquitinase ubiquitin aldehyde binding 1 (OTUB1) in human cancers can promote tumor progression by regulating the ferroptosis process in cancer cells [13]. Wan Seok Yang et al. showed that overexpression and knockdown of glutathione peroxidase 4 (GPX4) can modulate the lethality of 12 ferroptosis inducers, which indicated that GPX4 is an essential negative regulator of ferroptosis [14]. In addition, increasing evidence shows that various tumor cells are sensitive to ferroptosis. The induction of ferroptosis has emerged as a promising therapeutic alternative to trigger cancer cell death [15]. For instance, a study by Tesfayet al. [16] found that high expression of stearoyl CoA desaturase (SCD1) in ovarian cancer protected ovarian cancer cells from cell death, and the inhibition of SCD1 could promote ferroptosis both *in vitro* and *in vivo*, which provided a powerful new treatment for ovarian cancer. Moreover, adrenocortical carcinomas (ACCs) are characterized by poor survival. Alexia Belavgeni et al. found that the expression of GPX4 was significantly elevated in ACCs and that ACCs were more sensitive to ferroptosis. Thus, instead of traditional treatment with mitotane, it could be more effective to induce ferroptosis in ACC patients [17]. Thus, ferroptosis is a potential target for cancer therapy.

Moreover, with the utilization of bioinformatics techniques, researchers have developed some survival models based on ferroptosis-related genes for the prognostic prediction of cancer patients, including those with glioma [18], HCC [15] and clear cell renal cell carcinoma [19]. However, the role of ferroptosis-related genes in LUAD patients remains unknown. In the present study, we downloaded mRNA expression profiles and corresponding clinical data of LUAD patients from the TCGA database. Then, we constructed a prognostic multigene signature model including differentially expressed ferroptosis-related genes from the TCGA cohort, and the model was validated in the GSE30219 cohort. Finally, we analyzed the immune cell components of the tumor microenvironment to explore the underlying mechanisms of the difference in OS of individual LUAD patients.

## METHODS

### Data collection

RNA-seq expression data and clinical information of 510 LUAD tissues and 58 normal lung tissues were acquired from TCGA website. The RNA-seq expression profiles were normalized using the scale method provided in the “limma” R package. The GSE30219 dataset from the GEO database was used as the external validation cohort.

### Detection of FRGs

According to previously published studies, 60 ferroptosis-related genes were retrieved [9, 11, 20, 21]. The “limma” R package was used to identify differentially expressed FRGs between tumor tissues and adjacent normal tissues, with a false discovery rate (FDR) < 0.05 in the TCGA cohort. A total of 46 differentially expressed FRGs were identified in tumor tissues versus adjacent normal tissues on the basis of available mRNA expression data of LUAD from TCGA.

### Consensus clustering

By utilizing the “ConsensusClusterPlus” R package, LUAD patients from the TCGA dataset were split into two clusters in an unbiased and unsupervised manner [22]. To obtain a robust classification, the optimal number of clusters was further validated according to the total within sum of squares (WSS) and gap statistics. The differences in immune cells and the tumor immune microenvironment among the three clusters were compared by the K-W test or the Wilcoxon rank-sum test.

### Gene signature building and bioinformatics analysis

On the basis of the differentially expressed FRGs determined from the TCGA dataset, univariate Cox analysis was conducted in LUAD patients to screen survival-related FRGs, and FRGs with *P* values < 0.05 were retained. Finally, we conducted multivariate analysis to identify the optimal prognostic FRGs for the prognostic model. The risk scores of the LUAD patients were calculated based on the normalized gene expression levels and the Cox regression coefficients of the 5 selected FRGs. The formula was as follows: Risk score = e^sum (each gene’s expression × corresponding coefficient)^. A total of 501 LUAD patients were divided into high-risk and low-risk groups according to the median value of the risk score. By performing Kaplan–Meier survival analysis, we examined the survival of the two groups. Time-dependent receiver operating characteristic (ROC) curves were utilized to verify the prognostic performance of the model for overall survival (OS).

The immunoscore of each patient was calculated with the ESTIMATE algorithm in the R “estimate package.” The fraction of twenty-two immune cell types for each contained sample was yielded through cell type identification by estimating relative subsets of RNA transcripts (CIBERSORT; https://cibersortx.stanford.edu/). An algorithm with 1,000 permutations was adopted. Only samples with a CIBERSORT *p* < 0.05 were included for subsequent analysis of the differential immune infiltration levels among the subgroups grouped by clustering subtypes and risk scores.

The effects of CNAs of the 5 FRGs on the immune cell infiltration levels were evaluated by applying the Tumor Immune Estimation Resource (TIMER, https://cistrome.shinyapps.io/timer/), which consists of six immune cell types (i.e., B cells, CD8^+^ T cells, CD4^+^ T cells, macrophages, neutrophils, and dendritic cells). GISTIC 2.0 data were utilized in the TIMER.

### GSVA

Using the FRGs as reference genes and setting the *p* value < 0.05, we conducted GSVA to measure the signaling pathway variation score for clusters 1/2 by using the “GSVA” R package [23]. The enrichment score was calculated as the magnitude difference between the largest positive and negative random walk deviations.

### Experimental validation

To verify five FRGs expression profiles in LUAD and adjacent normal tissues, we conducted the experimental validation in 5 LUAD patients’ specimens who received operation at the First Affiliated Hospital of Wenzhou Medical University. The paired adjacent normal tissues were used as control. All procedures were performed in accordance with the ethical standards of the institutional and/or national research committee and with the Helsinki declaration, and approved by the Ethics Committee of the said hospital (Permit No. 2018014). Informed consent was obtained from all included patients.

Total RNA was extracted from paired LUAD tumor and normal tissues using TRIzol Reagent (Invitrogen) by following the manufacturer’s instructions (Invitrogen) and 1 μg of total RNA was used to perform reverse transcription with Prime Script RT reagent (TOYOBO). Then qRT-PCR was performed with SYBR Green Dye (Applied Biosystems) in triplicate and GAPDH was used as an internal control. Relative quantitation was calculated using the 2^-ΔΔCt^ method. The primers used in this study were as follows: CISD1 forward, 5′-AAGCTGTGTACTGCCGTTGT-3′ and reverse, 5′-CAGAGGGCCCACATTGTCTC-3′; NCOA4 forward, 5′-GAGGTGTAGTGATGCACGGAG-3′ and reverse, 5′-GACGGCTTATGCAACTGTGAA-3′; PHKG2 forward, 5′-AGGTCCATCATGCGGTCTCT-3' and reverse, 5′-AGTCGGATCTGCATATTGTCATC-3′; ACSL3 forward, 5′-ATGGAAAACCAACCTCATAGCAA-3′ and reverse, 5′-GCCATCCCAGTTATACCAGCAA-3′; PEBP1 forward, 5′-CTACACCTTGGTCCTGACAGA-3′ and reverse, 5′-GAGCCCACATAATCGGAGAGG-3′; GAPDH forward, 5′-CAGGGCTGCTTTTAACTCTGGTAA-3′ and reverse, 5′-GGGTGGAATCATATTGGAACATGT-3′.

### Statistical analysis

Statistical analysis was carried out using R version 3.6.1 The expression levels of FRGs in tumor and normal tissues were compared with one-way ANOVA. Survival curves were generated using the Kaplan–Meier method, and differences between groups were compared with the log rank test. All statistical tests were considered to be statistically significant at *p* < 0.05 (two-sided).

## RESULTS

### Identification of Ferroptosis-Related Genes (FRGs) in LUAD

**Figure 1:**
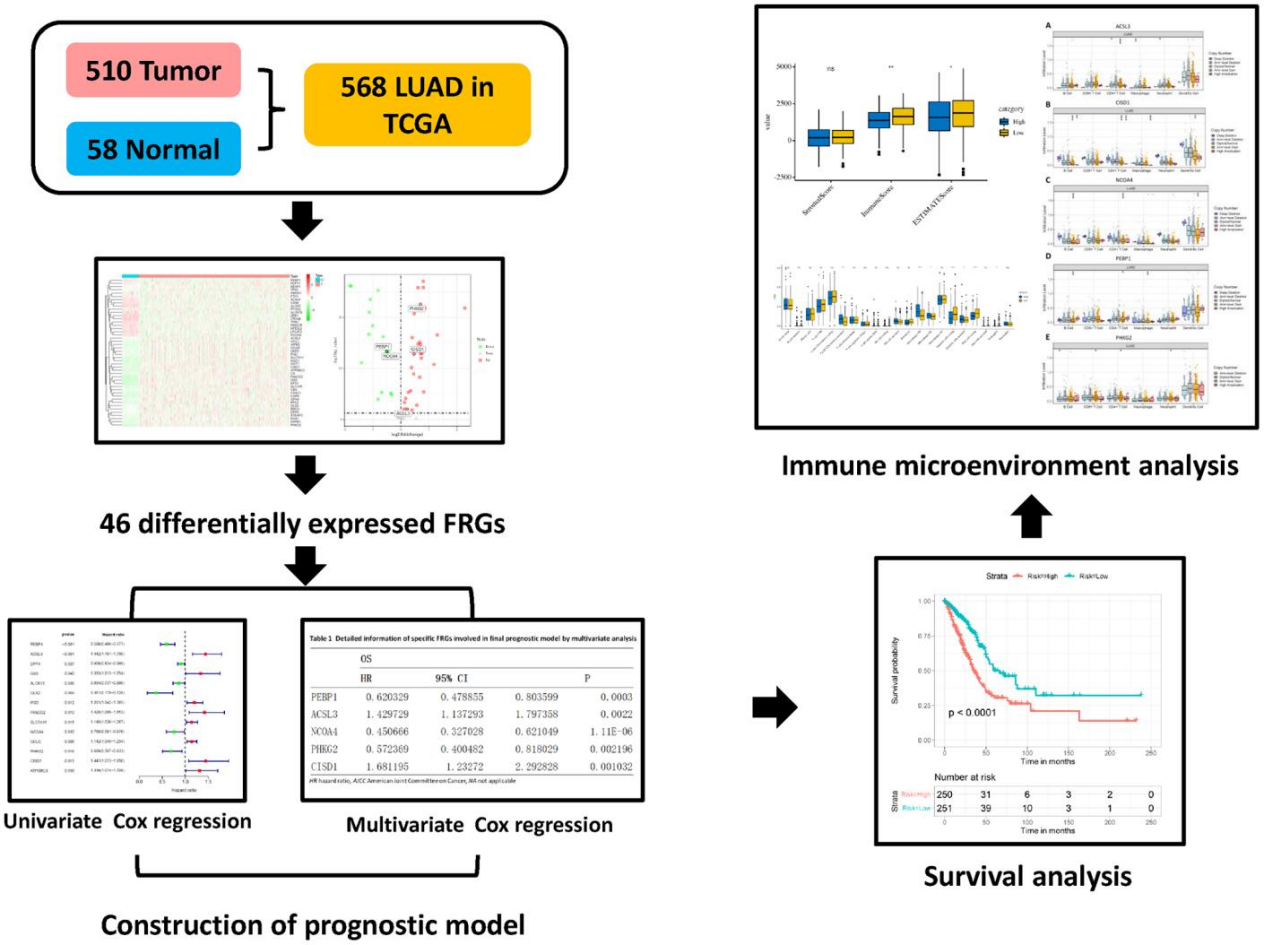
Flow chart of the data collection and analysis of FRGs in LUAD.

**Figure 2:**
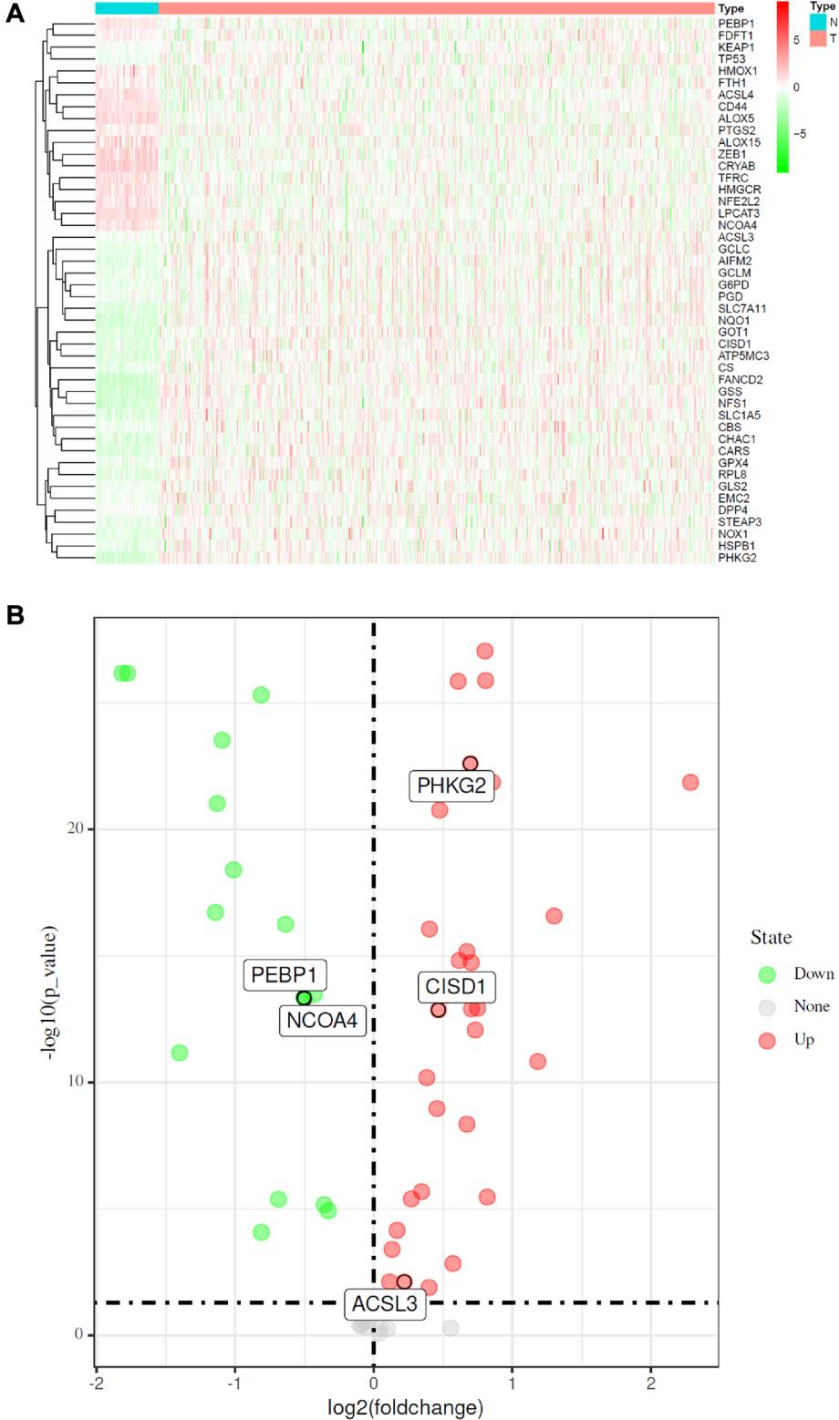
**Identification of FRGs in LUAD in the TCGA cohort.** (**A**) Heatmap of differentially expressed FRGs between 510 LUAD tissues and 58 normal adjacent tissues. (**B**) Volcano plot of the 46 differentially expressed FRGs identified in LUAD. The red and green points in the plot represent upregulated and downregulated FRGs, respectively.

First, the methods used for data collection and analysis of FRGs in LUAD are summarized in the flow chart shown in Figure 1. According to previous studies, we collected 60 ferroptosis-related genes (Supplementary Table 1). Then, to determine prognosis-specific FRGs for LUAD, we systematically analyzed and compared RNA-seq data of 510 LUAD tissues and 58 normal adjacent tissues from TCGA. Then, using the conditions of |log2fc|≥1 and FDR/adjusted *P* < 0.05, we identified a total of 46 differentially expressed FRGs (Supplementary Table 2). As shown in a heatmap (Figure 2A) and volcano plot (Figure 2B), different expression levels of FRGs between LUAD and normal tissues were evident.

### FRG-based clusters significantly associated with the survival and immune features of patients with LUAD

**Figure 3:**
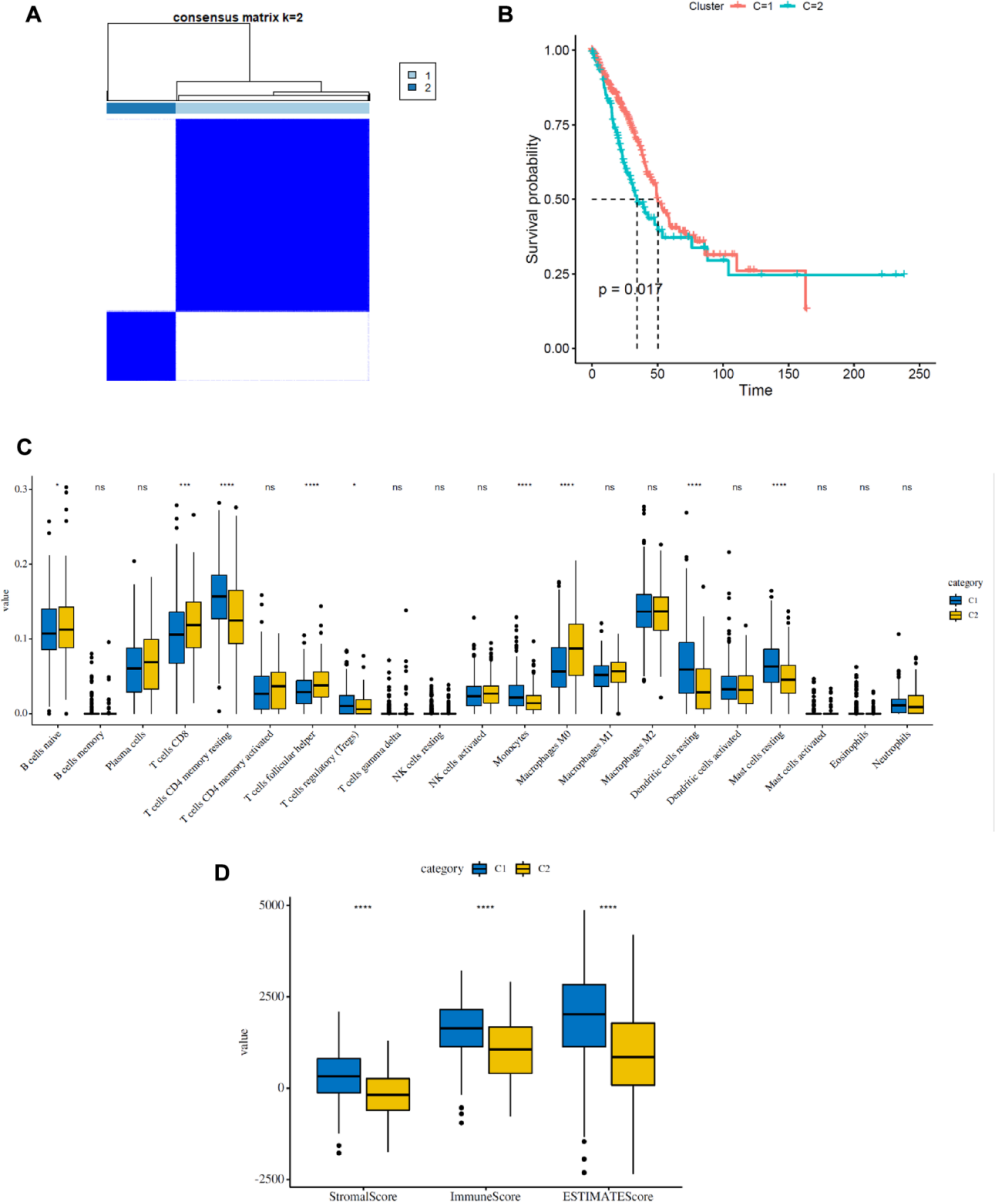
**Consensus clustering analysis of FRGs in LUAD.** (**A**) Consensus clustering matrix for k = 2. (**B**) Kaplan–Meier curves of the overall survival (OS) of patients with LUAD in two clusters (cluster 1/2) (*P* = 0.017). (**C**) The infiltrating levels of 21 immune cell types in two clusters (cluster 1/2). ^*^p < 0.05 and ^**^p < 0.01. (**D**) Immune score and stromal score of FRG-based clusters.

As shown in Figure 2A, the expression of FRGs was remarkably heterogeneous among LUAD patients. We suspect that variations in the expression of FRGs may be predictive of different clinical outcomes in individual patients. Thus, we performed unsupervised consensus analysis by using the “commonclusterplus” package. Based on the expression levels of FRGs from the TCGA database, LUAD patients were classified into two clusters (k = 2 was identified as the optimal clustering stability by testing k = 2 to 9; Figure 3A). The Kaplan–Meier curves showed that cluster 2 had significantly worse OS than cluster 1 (*P* = 0.017; Figure 3B). In the past few years, accumulating research has shown the potential association between ferroptosis and the TME, which is vital for the survival of cancer cells [5]. Given these findings, to investigate the effects of the expression of FRGs on the TME in LUAD, we evaluated the immune infiltrate level and immune and stromal scores of immune cells from the two clusters using the CIBERSORT and ESTIMATE computational methods. Subsequently, the fraction of 21 immune cell types of the two clusters were analyzed (Figure 3C). Cluster 1 showed higher infiltration levels of memory CD4 resting T cells, regulatory T cells (Tregs), monocytes, resting dendritic cells and resting mast cells, whereas cluster 2 showed higher infiltration levels of naive B cells, CD8 T cells, follicular helper T cells, and M0 macrophages. The ESTIMATE score was calculated as the sum of the immune score and stromal score. From Figure 3D, we observed that the immune score, stromal score and ESTIMATE score of cluster 1 were significantly higher than those of cluster 2, which suggested that the different TMEs in the two clusters might result in different survival of LUAD patients. Next, to elucidate the potential biological mechanisms resulting in the differences in the TME and OS between the two clusters, we performed GSVA (Supplementary Table 3). Heat maps showed that upregulation of the malignant hallmarks of tumors, including the Wnt/β-catenin signaling pathway, p53 pathway, KRAS signaling pathway, TGF-β signaling pathway, and PI3K/AKT/mTOR signaling pathway, was dynamically related to cluster 1 (Supplementary Figure 1). According to the above results, we can infer that these signaling pathways may be involved in the different TMEs of clusters 1 and 2. These results provide further support that variations in FRG expression affect the prognosis of LUAD patients.

### Construction and validation of the FRG-based prognostic model for LUAD patients in the TCGA and GSE30219 cohorts

**Figure 4:**
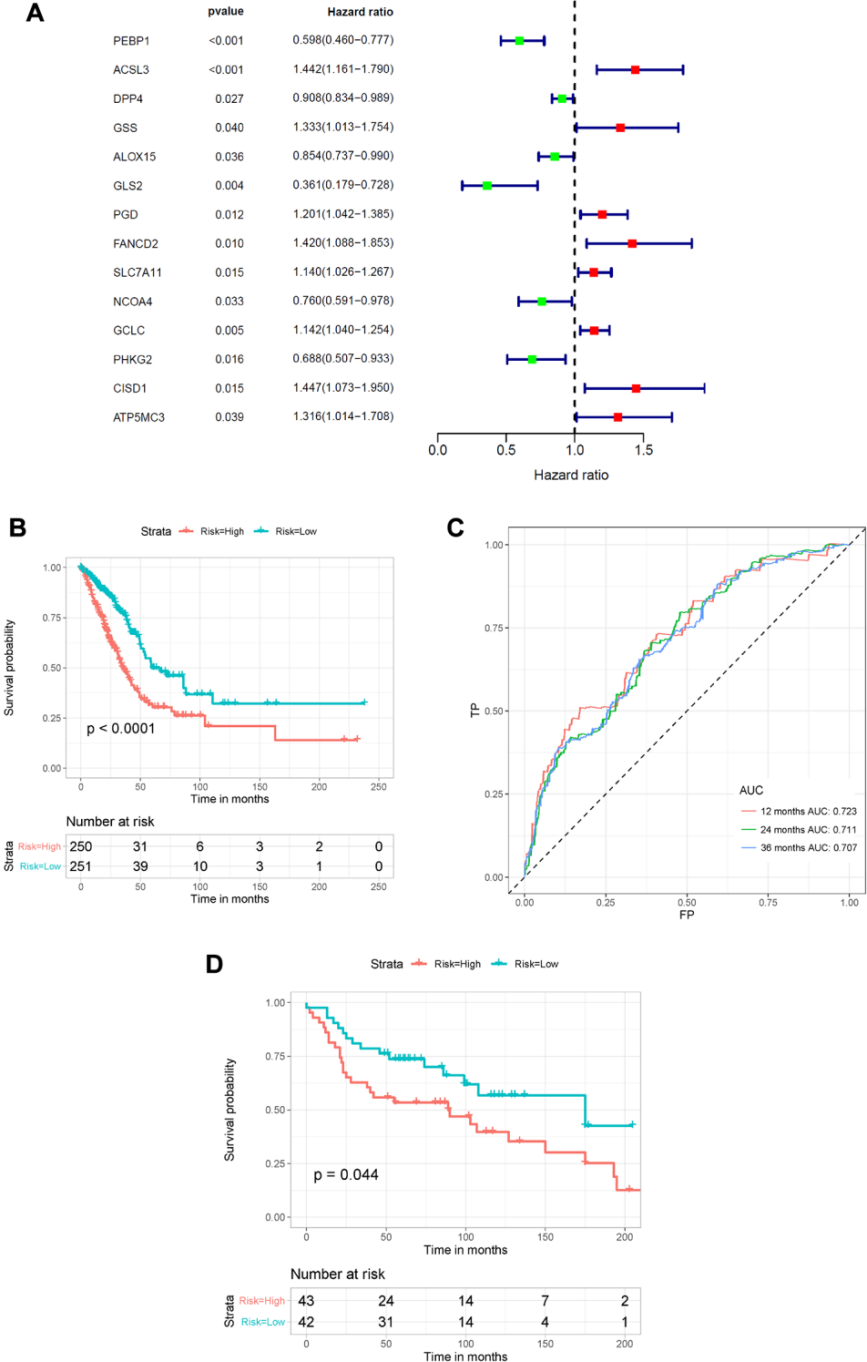
**Construction of the FRG-based survival model for prognostic prediction of LUAD.** (**A**) Univariate Cox regression analysis showing the hazard ratios (HRs) with 95% confidence intervals (CIs) and *p* values for 14 FRGs. (**B**) Kaplan–Meier survival curves showing the overall survival of high- and low-risk LUAD patients divided according to the risk score calculated using the new survival model based on the expression of 5 FRGs. (**C**) ROC curve analysis showing the prognostic prediction efficiency of the new survival model. (**D**) Kaplan–Meier survival curves analysis of the GSE30219 cohort.

To better elucidate the underlying relationship between FRGs and the prognosis of patients with LUAD, univariate Cox regression analysis of the expression of FRGs from the TCGA dataset was conducted. The results suggested that high expression of *ACSL3, GSS, PGD, FANCD2, SLC7A11, GCLC, CISD1*, and *ATP5MC3* was associated with worse survival rates of patients with LUAD compared to that of healthy individuals. However, high expression of *PEBP1, DPP4, ALOX15, GLS2, NCOA4* and *PHKG2* was correlated with better survival rates in LUAD patients (Figure 4A and Supplementary Table 4). Furthermore, multivariate regression analysis of these candidate FRGs showed that PEBP1, ACSL3, NCOA4, PHKG2, and CISD1 were independent prognostic factors for OS (Table 1). Then, based on the multivariate Cox regression results, we built a prognostic signature using the five candidate genes.

**Table 1:** Detailed information of specific FRGs involved in final prognostic model by multivariate analysis.

**Gene**	**Multivariate Cox regression analysis**				**coefficient**
	**HR**	**95% CI**		***P*-value**	
PEBP1	0.60412	0.465798	0.783518	0.000145	–0.50398
ACSL3	1.491651	1.137512	1.956042	0.003832	0.399883
NCOA4	0.433056	0.307214	0.610446	1.77E-06	–0.83689
PHKG2	0.54366	0.377357	0.783253	0.00107	–0.60943
CISD1	1.697829	1.227416	2.348532	0.001385	0.529351

Abbreviations: FRGs: ferroptosis-related genes; OS: overall survival; HR: hazard ratio; CI: confidence interval.

Using the risk scores calculated by the formula presented in the Methods section, LUAD patients were divided into a high-risk group (*n* = 250) and a low-risk group (*n* = 251) according to the median cutoff value. We used the Kaplan–Meier method and log-rank tests to explore the relationship between the risk score and the prognosis of LUAD patients. The results showed that patients in the high-risk group had a lower survival rate than that of patients in the low-risk group (*P* < 0.0001) (Figure 4B). Using ROC curve analysis, we determined the prognostic prediction efficiency of the survival model for LUAD patients. As shown in Figure 4C, the AUC was 0.723 at 1 year, 0.711 at 2 years, and 0.707 at 3 years. We then used the GSE30219 cohort to validate the predictive performance of the prognostic signature. Consistent with the above results, patients with LUAD in the high-risk group had reduced survival compared with that of patients in the low-risk group (Figure 4D, *P* = 0.044). In addition, the AUC of the 5-gene signature was 0.617 at 1 year, 0.603 at 2 years, and 0.557 at 3 years (Supplementary Figure 2).

### Effects of genetic alterations of the FRG-based signatures on immune cell infiltration

**Figure 5:**
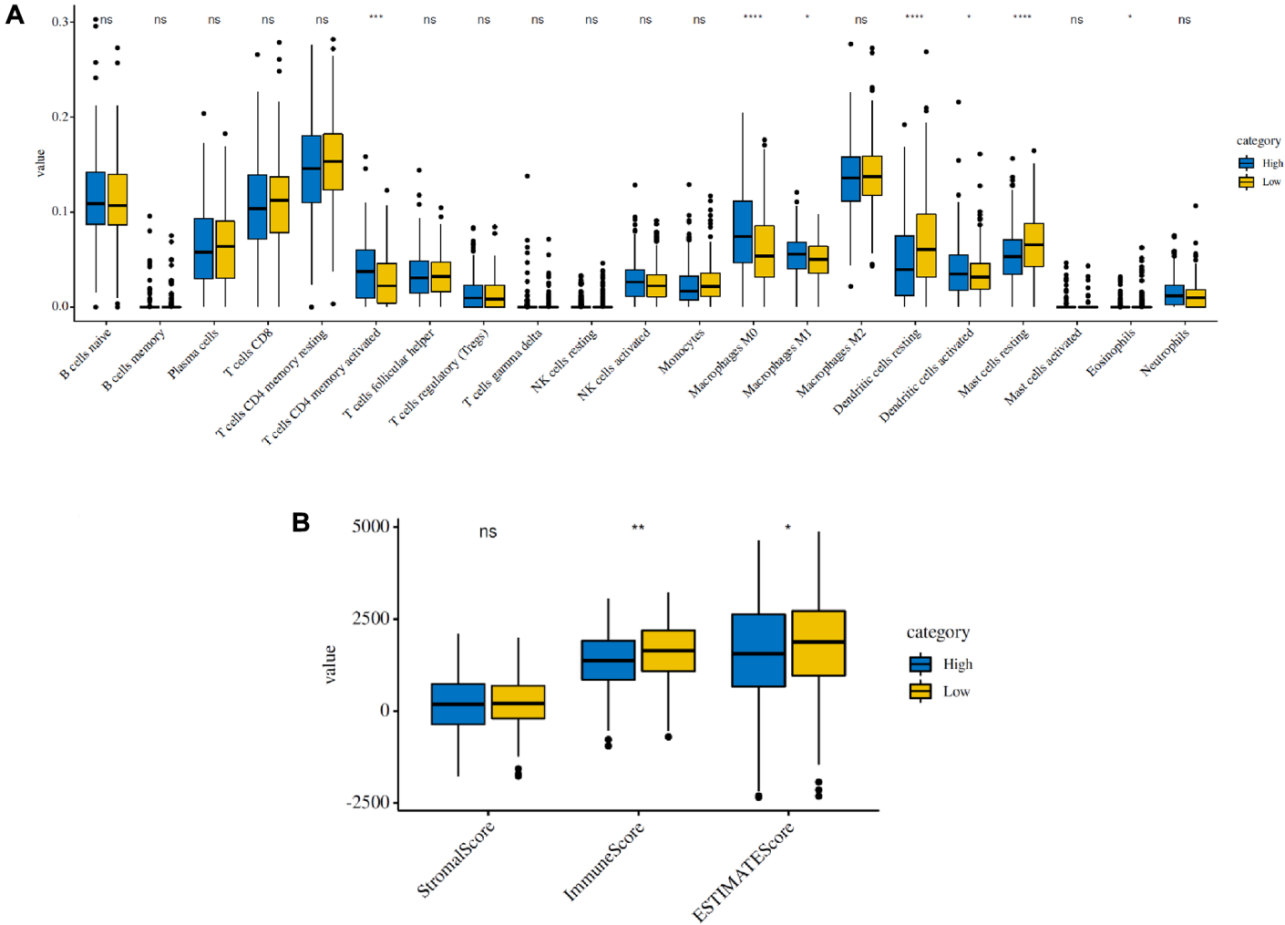
**Immune infiltration in the high-risk and low-risk groups in the TCGA cohort.** (**A**) The infiltrating levels of 21 immune cell types in the two groups (cluster 1/2). ^*^p < 0.05 and ^**^p < 0.01. (**B**) Immune score and stromal score of the two groups.

To estimate the effects of the 5 FRG-based signatures on the LUAD immune microenvironment, the relationship between the risk score and the infiltration of different immune cell types was further explored. Subsequently, the fraction of 21 immune cell types were analyzed and compared between the high-risk and low-risk groups (Figure 5A). We found that four kinds of immune cells showed higher infiltration levels in the high-risk group, including CD4 memory-activated T cells, M0 macrophages, M1 macrophages and activated dendritic cells, and three kinds of immune cells showed higher infiltration levels in the low-risk group, including resting mast cells, activated mast cells and eosinophils. Then, based on the ESTIMATE algorithm, we calculated the immune and stromal scores. The results showed that the high-risk group had higher immune and stromal scores than those of the low-risk group (Figure 5B). These results confirmed that FRG-based risk signatures were implicated in the LUAD immune microenvironment.

**Figure 6:**
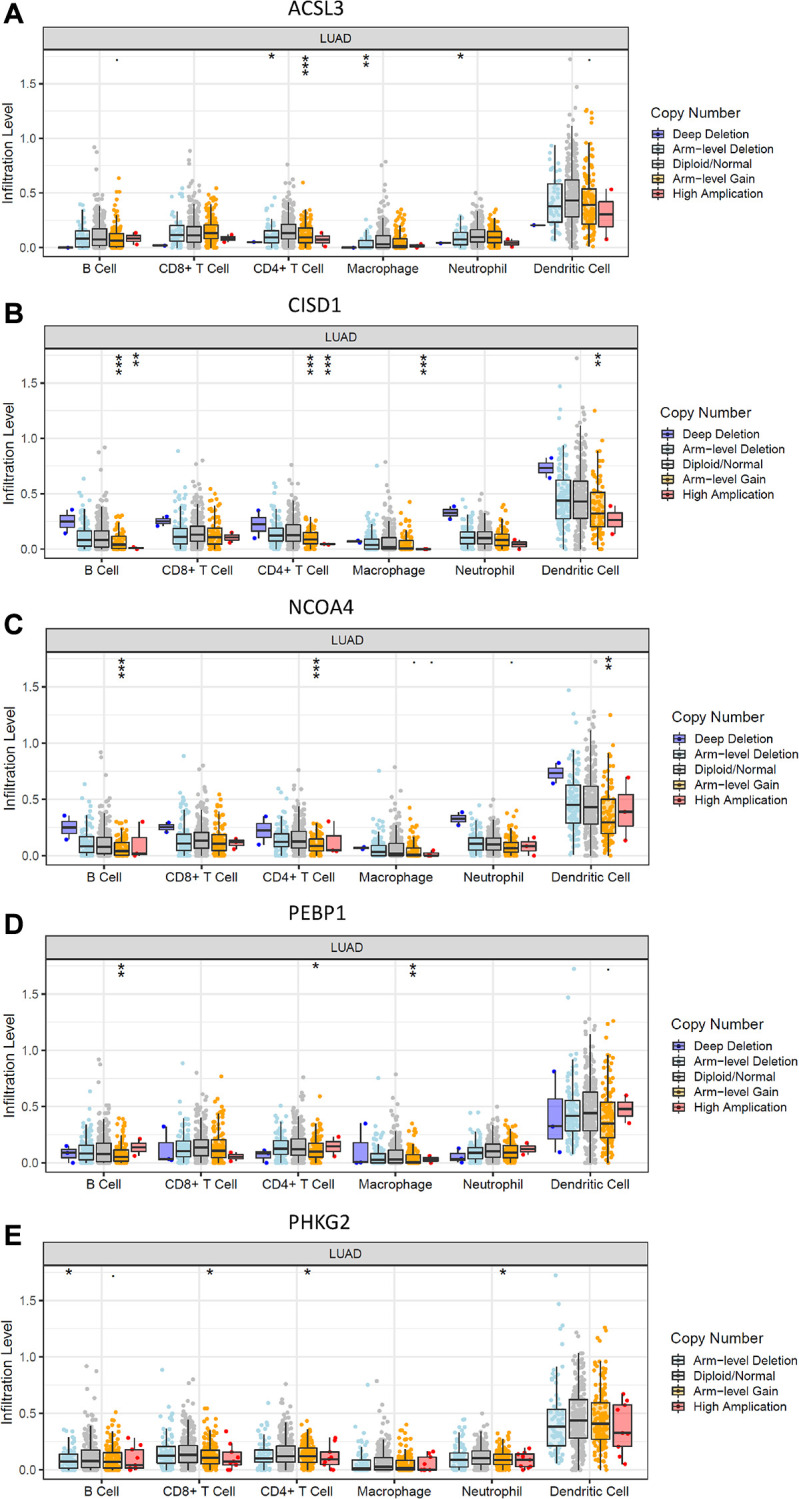
**Effects of genetic alterations of FRG-relevant signatures on immune cell infiltration.** (**A**–**E**) ACSL3 (**A**), CISD1 (**B**), NCOA4 (**C**), PEBP1 (**D**) and PHKG2 (**E**). ^*^p < 0.05, ^**^p < 0.01, and ^***^p < 0.001.

To elucidate the underlying mechanisms by which the risk score was related to different immune cell infiltrations, the effects of somatic cell copy number alterations (CNAs) of the five FRG-based signatures on immune cell infiltration were further analyzed. We observed that the infiltration levels of B cells, CD8^+^ T cells, CD4^+^ T cells, macrophages, neutrophils and dendritic cells in the TME in LUAD patients were obviously influenced by arm-level deletion and arm-level gain of the five identified FRGs-based signature, which further demonstrated that the five FRGs played an important role in the regulation of the TME in LUAD patients (Figure 6).

### Clinical experimental validation

**Figure 7:**
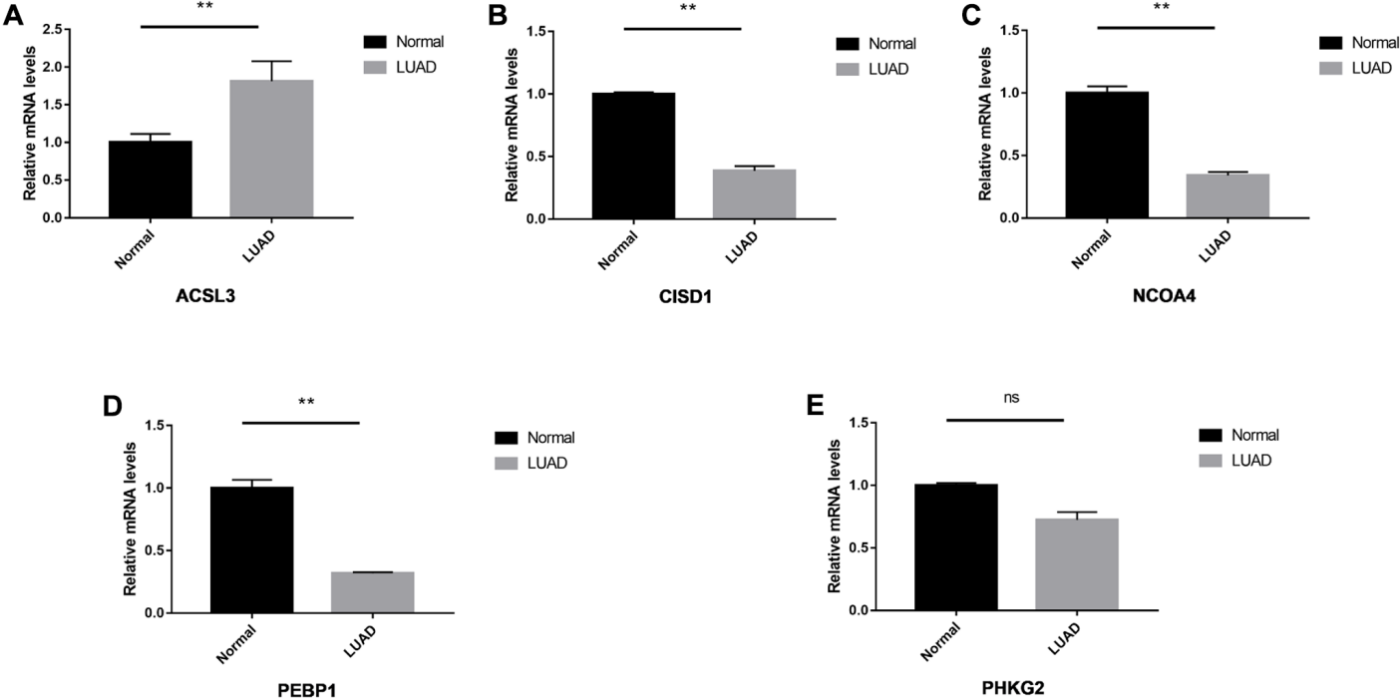
**The relative expression levels of the five genes in normal and LUAD tissues.** The ACSL3 (**A**) was up-regulated significantly and CISD1 (**B**), NCOA4 (**C**) and PEBP1 (**D**) were down-regulated in the LUAD tissues. No significant differences were observed in the PHKG2 (**E**). ^*^P < 0.05; ^**^P < 0.01, ns: not significant.

We performed the validation in clinical specimens following the steps described in the Method. We verified the five FRGs (PEBP1, ACSL3, NCOA4, PHKG2, CISD1) which constructed the survival model of LUAD patients. As the PCR results showed ACSL3 was up-regulated and the PEBP1, CISD1 and NCOA4 were significantly down-regulated in the LUAD tissues. There was no statistical significance in the expression of PHKG2 between the normal and LUAD specimens. The details of the five genes were visualized in Figure 7A–7E.

## DISCUSSION

It is well known that lung cancer is responsible for more deaths than any other type of cancer worldwide. NSCLC accounts for almost 80% of lung cancer patients, approximately 50% of whom have LUAD [24]. The survival rate of LUAD patients remains poor despite improvements have been made in therapeutic regimens. The complex etiologic factors, along with the high-level heterogeneity of LUAD, make the prognostic prediction challenging. Therefore, there is an urgent need to develop novel prognostic models.

Increasing evidence has shown that ferroptosis, an iron-dependent type of regulated cell death, plays a crucial role in tumorigenesis and cancer therapeutics. However, the profile of the effect of ferroptosis in LUAD has yet to be clarified. In our study, we found that most FRGs (46/60) were differentially expressed between LUAD tumor tissue and adjacent normal tissue. On the basis of univariate and multivariate Cox regression analyses, we constructed a novel prognostic model that included 5 FRGs from the TCGA database. The FRG-based signature was well validated in an external cohort. These results indicated the crucial role of ferroptosis in LUAD and confirmed that the FRG-based prognostic model proposed in this study could improve survival predictions of patients with LUAD.

The prognostic model proposed in the present study is composed of 5 ferroptosis-related genes (PEBP1, ACSL3, NCOA4, PHKG2, CISD1). Ferroptosis is a complicated metabolic process involving ROS, iron, and PUFAs. The genes associated with these processes can modulate sensitivity to ferroptosis. A comprehensive summary of the genes and pathways involved in ferroptosis-related metabolism was conducted by Behrouz Hassannia et al. in 2019. NCOA4, CISD1, and PHKG2 were related to iron metabolism, and ACSL3 and PEBP1 were associated with lipid metabolism [11].

Phosphatidylethanolamine-binding protein 1 (PEBP1), a scaffold protein inhibitor that binds to the two isoforms of 15-lipoxygenase (15-LO), can promote ferroptosis in asthma, kidney injury, and brain trauma [25]. Acyl-coenzyme A synthetase long-chain family member 3 (ACSL3), a fatty acid-activating enzyme, participates in activating exogenous monounsaturated fatty acids (MUFAs) by transforming them into fatty acyl-CoAs, which can promote a ferroptosis-resistant cell state by suppressing lipid ROS accumulation [26]. Nuclear receptor coactivator 4 (NCOA4) is known to be a selective cargo receptor for the selective autophagic turnover of ferritin in ferroptosis [27]. A study conducted by Wen Hou et al. demonstrated that overexpression of NCOA4 increased ferritin degradation and thus promoted ferroptosis by increasing iron levels, resulting in oxidation [28]. Similarly, Gao and colleagues [29] reported that NCOA4 contributed to ferroptosis by regulating cellular iron and accumulating cellular ROS. Phosphorylase kinase G2 (PHKG2) encodes the catalytic subunit of the phosphorylase kinase (PHK) complex. Knockdown of PHKG2 can influence the level of ROS or affect cellular iron homeostasis, leading to a reduction in lipid peroxidation upon erastin treatment [30]. CISD1, a mitochondrial protein located in the outer membrane, has been shown to negatively regulate erastin-induced ferroptosis in HCC by limiting mitochondrial iron uptake and therefore suppress ferroptosis [12, 15]. Recent studies also revealed that high expression of CISD1 contributes to the growth of breast cancer cells by mediating iron and reactive oxygen homeostasis in mitochondria [31–33], which is believed to be a promising target for cancer therapy. In conclusion, previous studies have reported that PHKG2, PEBP1 and NCOA4 are positive regulators that promote ferroptosis in some kinds of cancers, whereas the remaining two genes (ACSL3 and CISD1) suppress ferroptosis in cells. However, very little research has addressed the relationship these five ferroptosis genes with LUAD. In our prognostic model of LUAD, we surprisingly found that ACSL3 and CISD1 were promoters of ferroptosis, whereas the remaining three genes were suppressors of ferroptosis, which was opposite of the results acquired in other cancers. Whether these genes play a role in the prognosis of LUAD patients by influencing ferroptosis remains to be elucidated.

The tumor microenvironment (TME) mainly functions as “fertile soil” for the growth of cancer cells [34]. In the past few years, accumulating research has shown the potential association between ferroptosis and the TME, which is vital for the survival of cancer cells [35]. The TME is abundant with different types of immune cells, such as tumor-associated macrophages (TAMs), NK cells, and T cells and so on, are critical for the maintenance of iron homeostasis. Stefaniaet al. [36] found that M2 macrophages could disrupt iron homeostasis in cancer cells due to their iron-releasing properties, which influenced the survival of cancer cells. Thus, iron metabolism in M2 macrophages may provide a potential therapeutic target for suppressing tumor growth. In addition, it has been reported that increased Tregs or macrophages are related to poor prognosis of patients with hepatocellular cancer [37, 38]; similarly, these two types of immune cells were also found to be increased in LUAD patients in the high-risk group in our research (Figure 5A). Another study also found that Th1 cells, natural killer T cells and monocytes were involved in the maintenance of iron homeostasis [39]. Therefore, the regulation of ferroptosis may present us with a new therapeutic opportunity to treat cancer. Recently, a study demonstrated that the TME played a crucial regulatory role in the initiation and progression of LUAD [5], which might be responsible for its heterogeneity, leading to diverse clinical outcomes and therapeutic responses in LUAD patients [40, 41]. The results of Bi et al. implied that the immunoscore and immune cell infiltration levels in the TME influenced the survival of LUAD patients, which might provide novel insight into overcoming the problem of making survival predictions of LUAD patients using the TME [5].

In the past few years, antitumor immunotherapy has drawn increasing attention and achieved considerable success in the clinic. In particular, immune checkpoint blockade has revolutionized cancer treatment [42]. Emerging evidence has revealed the strong relationship between ferroptosis and tumor immunity [43, 44]. A study published in Nature by Wang et al. [45] first demonstrated that activated CD8^+^ T cells could play an important role in antitumor immunotherapy by initiating ferroptosis in cancer cells. Mechanistically, the authors confirmed that interferon gamma (IFN-γ) released from activated CD8^+^ T cells impaired the uptake of cystine by cancer cells, therefore enhancing lipid peroxidation and promoting ferroptosis [46], which offers a new direction for cancer immunotherapy from a ferroptosis perspective. Likewise, *in vitro*, Cao and colleagues identified that intracellular accumulation of oxidized lipids in tumor-associated dendritic cells (DCs) impaired the ability of DCs to present antigens, thus leading to dysfunction of CD8^+^ T cells in triggering an immune response. As a result, we can infer that ferroptosis of cancer cells may be regulated by CD8^+^ T cells and DCs through oxidized lipids and PUFAs within cells, which suggests future potential therapeutic avenues [47, 48]. Together, these new findings provide new insight into ferroptosis as a potential target for cancer immunotherapy.

Inevitably, there are several limitations in our study. First, based on retrospective data from the TCGA database, we constructed a survival model based on FRGs for making prognostic predictions of LUAD patients. Validation of the model was performed using retrospective data from the GSE30219 cohort. Thus, we need more prospective data to verify the clinical application value of our FRG-based survival model. Second, there are a lot of excellent methods to perform the regression analysis better than lasso, especially the network-regularized regression method [49], it is a drawback of our study that we didn’t take such an approach. In addition, we did not perform experiments to investigate the molecular mechanism underlying the 5 identified ferroptosis-related genes (PEBP1, ACSL3, NCOA4, PHKG2, CISD1) and their effects on the development and survival of LUAD patients. Further studies that include molecular mechanism experiments are required to elucidate the relationship between the risk score and immune activity of LUAD.

## CONCLUSIONS

In conclusion, a novel prognostic model of 5 ferroptosis-related genes was constructed in this study. This model was shown to be independently associated with OS in both the TCGA and GSE30219 cohorts, providing a candidate model for predicting survival of LUAD patients. Our study may provide insight into the identification of therapeutic targets for LUAD.

## Supplementary Materials

Supplementary Figures

Supplementary Tables 1-3

Supplementary Table 4
