# Supplementary material for: A novel ferroptosis-related gene signature for prognostic prediction of patients with lung adenocarcinoma
Source: Aging (Albany NY). 2021 Jun 11;13(12):16144–64. doi: 10.18632/aging.203140 (PMC8266333; doi:10.18632/aging.203140)
Supplement: Supplementary Figures [file aging-13-203140-s001.pdf]

SUPPLEMENTARY FIGURES

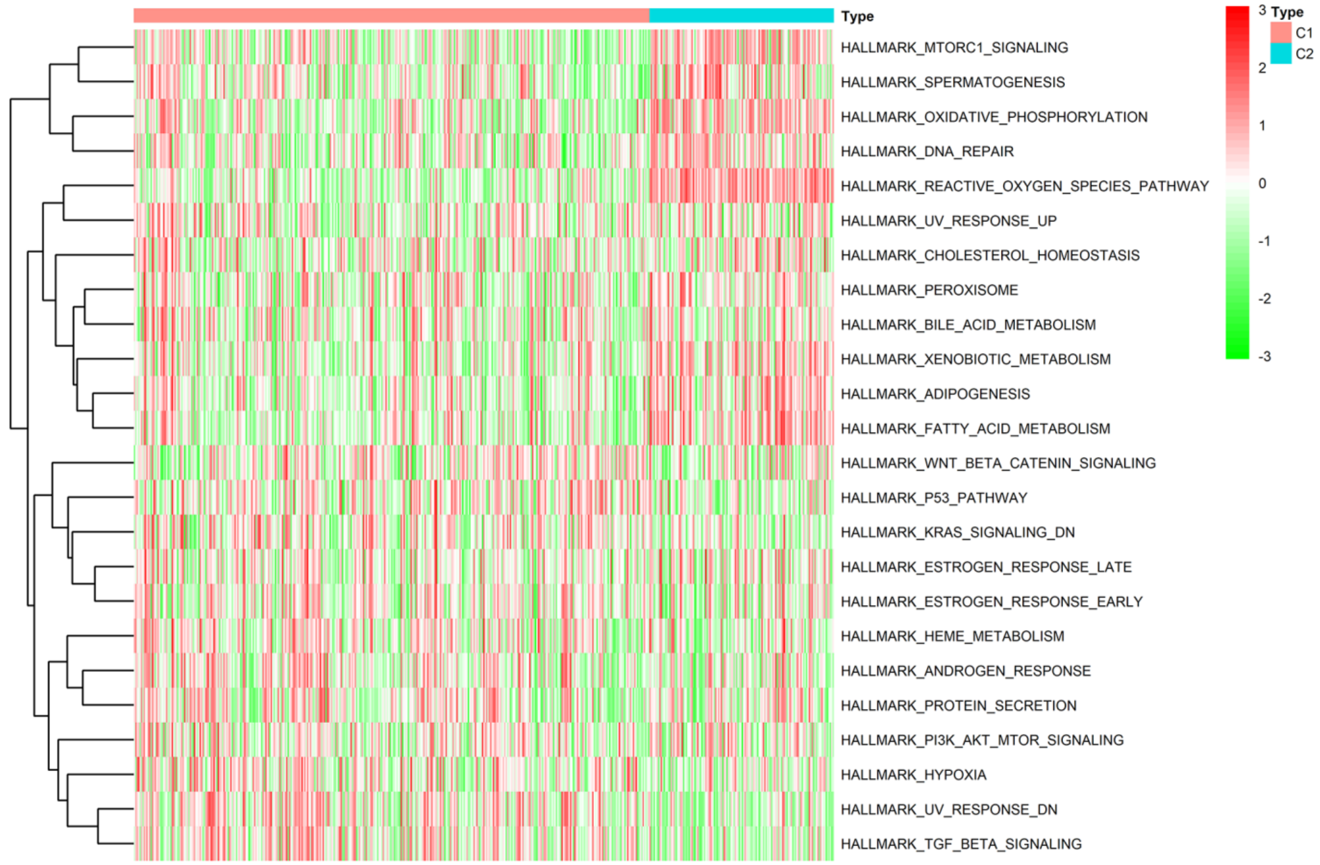

Supplementary Figure 1. Heatmaps of the GSVA results of two clusters (clusters 1/2) in the TCGA cohort.

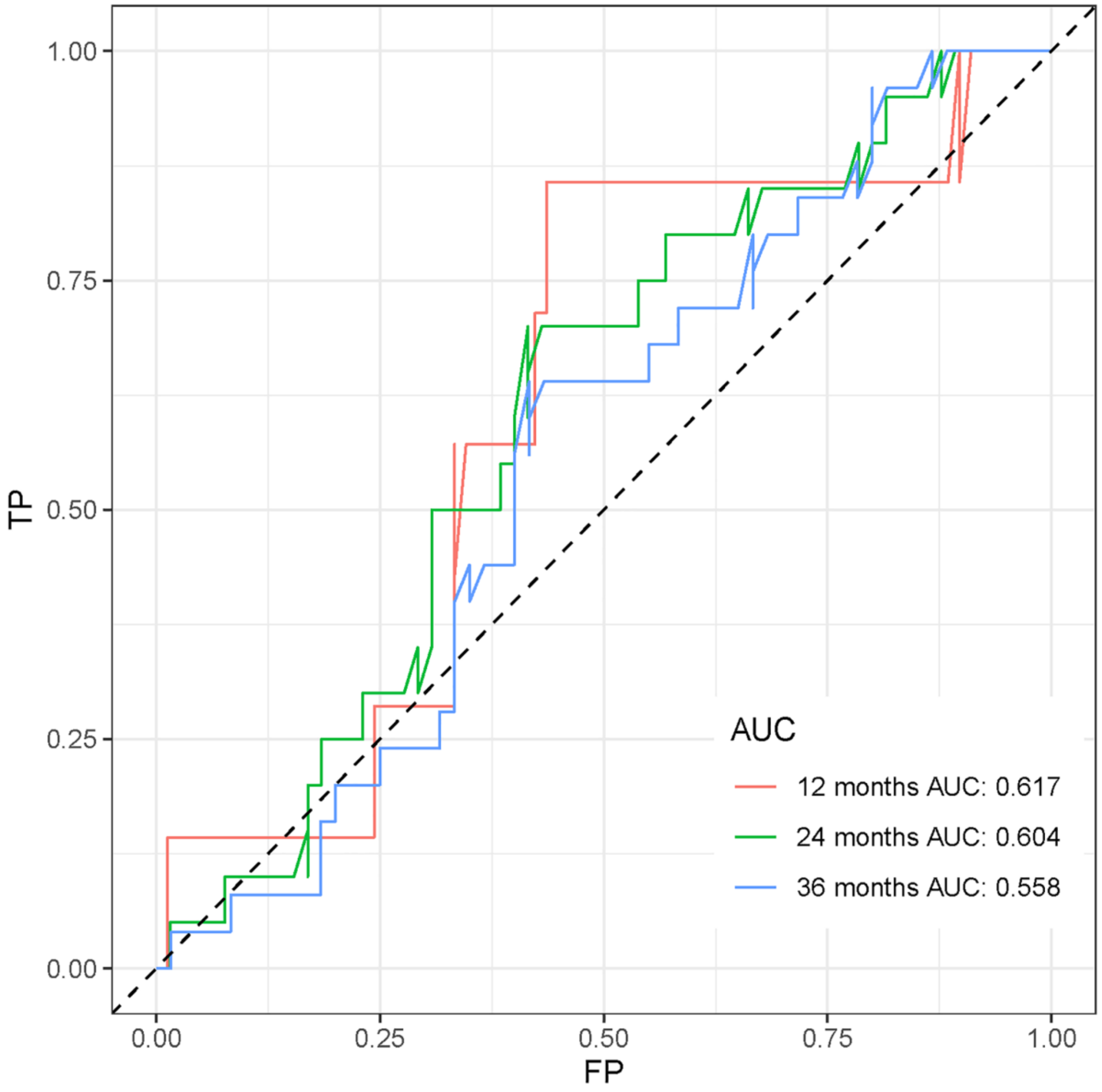

Supplementary Figure 2. The ROC curve analysis of the GSE30219 cohort.
