# Supplementary material for: A novel ferroptosis-related gene signature for prognostic prediction of patients with lung adenocarcinoma
Source: Aging (Albany NY). 2021 Jun 11;13(12):16144–64. doi: 10.18632/aging.203140 (PMC8266333; doi:10.18632/aging.203140)
Supplement: Supplementary Tables 1-3 [file aging-13-203140-s002.pdf]

SUPPLEMENTARY TABLES

Supplementary Table 1. There were 60 FRGs identified from previous literature.

ACSL4
AKR1C1
AKR1C2
AKR1C3
ALOX15
ALOX5
ALOX12
ATP5MC3
CARS
CBS
CD44
CHAC1
CISD1
CS
DPP4
FANCD2
GCLC
GCLM
GLS2
GPX4
GSS
HMGCR
HSPB1
CRYAB
LPCAT3
MT1G
NCOA4
PTGS2
RPL8
SAT1
SLC7A11
FDFT1
TFRC
TP53
EMC2
AIFM2
PHKG2
HSBP1
ACO1
FTH1
STEAP3
NFS1
ACSL3
ACACA
PEBP1

ZEB1
SQLE
FADS2
NFE2L2
KEAP1
NQO1
NOX1
ABCC1
SLC1A5
GOT1
G6PD
PGD
IREB2
HMOX1
ACSF2

Supplementary Table 2. A total of 46 differentially expressed FRGs identified between the 510 LUAD tissues and 58 normal adjacent tissues with adj *P* value < 0.05.

Gene	Con Mean	Treat Mean	Log FC	*P* Value	Fdr
LPCAT3	3.159412	2.3466	–0.81281	5.02E-27	4.77E-26
GPX4	6.544112	6.888866	0.344754	1.23E-06	2.06E-06
G6PD	4.257216	5.075513	0.818296	2.09E-06	3.40E-06
CBS	0.092336	0.223733	0.131396	0.00029	0.000394
PEBP1	7.485216	6.98014	–0.50508	1.77E-14	4.38E-14
ACSL3	3.739216	3.959468	0.220251	0.006011	0.007614
DPP4	3.488111	4.058837	0.570726	0.001083	0.001435
CD44	6.034208	4.905438	–1.12877	1.81E-22	9.36E-22
GSS	4.147503	4.954165	0.806662	9.01E-28	1.28E-26
ALOX15	2.358251	0.957525	–1.40073	3.43E-12	6.74E-12
GLS2	0.207865	0.322023	0.114158	0.005949	0.007614
RPL8	8.804998	9.509746	0.704748	5.57E-14	1.22E-13
PGD	5.81347	6.212381	0.398911	0.010224	0.012669
TFRC	5.22247	4.080503	–1.14197	4.60E-18	1.87E-17
CHAC1	1.018744	1.721919	0.703175	6.37E-16	1.82E-15
FDFT1	4.921858	4.564213	–0.35765	4.53E-06	6.80E-06
FANCD2	0.702023	1.504381	0.802358	1.53E-29	8.74E-28
EMC2	3.38608	3.656837	0.270758	2.52E-06	4.00E-06
ZEB1	2.862145	1.767656	–1.09449	3.60E-25	2.94E-24
ACSL4	4.86119	3.849839	–1.01135	9.01E-20	3.95E-19
ALOX5	5.421907	3.605919	–1.81599	2.39E-28	6.67E-27
SLC7A11	0.538602	1.839077	1.300475	6.91E-18	2.62E-17
AIFM2	2.175042	2.791287	0.616244	5.04E-16	1.51E-15
KEAP1	4.090052	4.257769	0.167717	4.89E-05	6.96E-05

HMGCR	3.841814	3.205199	–0.63662	1.57E-17	5.59E-17
CRYAB	3.704674	1.928149	–1.77652	3.51E-28	6.67E-27
NCOA4	6.144397	5.644094	–0.5003	2.02E-14	4.79E-14
HMOX1	5.277925	4.590963	–0.68696	2.69E-06	4.14E-06
STEAP3	3.333276	4.064916	0.73164	4.13E-13	8.42E-13
NQO1	3.429814	5.714839	2.285025	2.38E-23	1.36E-22
GCLC	1.880546	3.063667	1.183121	7.79E-12	1.48E-11
NFS1	1.777912	2.386447	0.608535	1.21E-27	1.38E-26
FTH1	8.465463	8.137851	–0.32761	8.08E-06	1.18E-05
PTGS2	4.179196	3.367837	–0.81136	5.98E-05	8.31E-05
TP53	3.562903	4.018418	0.455515	6.00E-10	1.07E-09
NOX1	0.374526	0.776181	0.401655	2.56E-17	8.57E-17
SLC1A5	4.795671	5.466592	0.670922	2.12E-16	6.70E-16
GOT1	3.664455	4.52046	0.856006	2.28E-23	1.36E-22
CS	4.339807	4.722226	0.382419	3.50E-11	6.44E-11
HSPB1	7.181479	7.930812	0.749333	5.05E-14	1.15E-13
PHKG2	1.850388	2.547034	0.696646	3.47E-24	2.47E-23
CISD1	2.683009	3.149152	0.466143	6.42E-14	1.35E-13
NFE2L2	4.857844	4.427938	–0.42991	1.21E-14	3.27E-14
ATP5MC3	3.952662	4.4678	0.515138	1.36E-14	3.52E-14
CARS	2.234765	2.709365	0.4746	3.62E-22	1.72E-21
GCLM	2.483958	3.155553	0.671595	2.59E-09	4.47E-09

Supplementary Table 3. The GSVA results in two clusters (cluster1/2) in TCGA dataset.

Id	Log FC	Ave Expr	T	*P* Value	adj.*P*.Val	B
HALLMARK_REACTIVE_OXYGEN_SPECIES_PATHWAY	–0.12439	–0.04013	–12.5544	1.17E-31	5.85E-30	60.98507
HALLMARK_ADIPOGENESIS	–0.09228	–0.06732	–11.1868	4.15E-26	2.03E-24	48.31816
HALLMARK_FATTY_ACID_METABOLISM	–0.0992	–0.05793	–10.4683	2.41E-23	1.16E-21	42.0136
HALLMARK_XENOBIOTIC_METABOLISM	–0.07355	–0.0452	–8.61505	8.97E-17	4.22E-15	27.06837
HALLMARK_CHOLESTEROL_HOMEOSTASIS	–0.08923	–0.0809	–8.59963	1.01E-16	4.64E-15	26.95273
HALLMARK_HEME_METABOLISM	–0.06282	–0.07123	–8.35957	6.14E-16	2.76E-14	25.17307
HALLMARK_PEROXISOME	–0.06174	–0.04912	–8.14164	3.06E-15	1.35E-13	23.59048
HALLMARK_PI3K_AKT_MTOR_SIGNALING	–0.06342	–0.06412	–7.57823	1.68E-13	7.25E-12	19.64987
HALLMARK_UV_RESPONSE_UP	–0.05034	–0.04669	–7.55563	1.97E-13	8.27E-12	19.49645
HALLMARK_ESTROGEN_RESPONSE_LATE	–0.05688	–0.05315	–7.42486	4.83E-13	1.98E-11	18.61592
HALLMARK_BILE_ACID_METABOLISM	–0.07118	–0.06607	–7.4066	5.47E-13	2.19E-11	18.49392
HALLMARK_ANDROGEN_RESPONSE	–0.07252	–0.08439	–6.71009	5.23E-11	2.04E-09	14.02504
HALLMARK_OXIDATIVE_PHOSPHORYLATION	–0.0925	–0.02326	–6.29895	6.52E-10	2.48E-08	11.56034
HALLMARK_ESTROGEN_RESPONSE_EARLY	–0.05285	–0.05997	–5.70771	1.95E-08	7.23E-07	8.251375
HALLMARK_MTORC1_SIGNALING	–0.06535	–0.01653	–5.27857	1.94E-07	6.97E-06	6.029441
HALLMARK_P53_PATHWAY	–0.041	–0.05822	–4.66923	3.88E-06	0.000136	3.143607

HALLMARK_SPERMATOGENESIS	–0.0423	–0.01905	–4.6555	4.14E-06	0.000141	3.08229
HALLMARK_PROTEIN_SECRETION	–0.05754	–0.05715	–4.39619	1.34E-05	0.000443	1.95559
HALLMARK_DNA_REPAIR	–0.03859	–0.00917	–4.11694	4.48E-05	0.001435	0.809281
HALLMARK_UV_RESPONSE_DN	–0.0528	–0.09663	–4.04061	6.16E-05	0.00191	0.508204
HALLMARK_HYPOXIA	–0.03702	–0.05444	–3.3353	0.000915	0.027441	–2.02172
HALLMARK_TGF_BETA_SIGNALING	–0.04466	–0.08957	–3.20737	0.001424	0.041309	–2.43114
HALLMARK_KRAS_SIGNALING_DN	–0.02526	–0.03956	–3.16511	0.001644	0.046023	–2.56298
HALLMARK_WNT_BETA_CATENIN_SIGNALING	–0.03848	–0.04962	–3.14145	0.00178	0.04805	–2.63608

Please browse Full Text version to see the data of Supplementary Table 4.

Supplementary Table 4. The results of the univariate Cox regression analysis.
